# Effects of Acupuncture on Delayed-Onset Muscle Soreness: A Systematic Review and Meta-Analysis

**DOI:** 10.1155/2020/5864057

**Published:** 2020-06-27

**Authors:** Wen-Dien Chang, Nai-Jen Chang, Hung-Yu Lin, Jih-Huah Wu

**Affiliations:** ^1^Department of Sport Performance, National Taiwan University of Sport, Taichung, Taiwan; ^2^Department of Sports Medicine, Kaohsiung Medical University, Kaohsiung, Taiwan; ^3^Department of Occupational Therapy, Asia University, Taichung, Taiwan; ^4^Department of Biomedical Engineering, Ming Chuan University, Taoyuan, Taiwan

## Abstract

**Objectives:**

Evidence for the effects of acupuncture on delayed-onset muscle soreness (DOMS) is inconsistent. The aim of this study was to explore the effects of acupuncture on DOMS.

**Methods:**

Studies investigating the effect of acupuncture on DOMS in humans that were published before March 2020 were obtained from eight electronic databases. The affected muscles, groups, acupuncture points, treatment sessions, assessments, assessment times, and outcomes of the included articles were reviewed. The data were extracted and analyzed via a meta-analysis.

**Results:**

A total of 15 articles were included, and relief of DOMS-related pain was the primary outcome. The statistical meta-analysis showed that there were no significant differences between acupuncture and sham/control groups, except for acupuncture for DOMS on day 1 (total SMD = −0.62; 95% CI = −1.12∼0.11, *P* < 0.05) by comparing with control groups.

**Conclusion:**

Acupuncture for DOMS exhibited very-small-to-small and small-to-moderate effects on pain relief for the sham and no acupuncture conditions, respectively. Evidence indicating the effects of acupuncture on DOMS was little because the outcome data during the follow-up were insufficient to perform an effective meta-analysis.

## 1. Introduction

Delayed-onset muscle soreness (DOMS) is a common myogenic pain that occurs after strenuous exercise training, especially after eccentric muscle contraction exercise [1]. The clinical signs of DOMS include muscle soreness, tenderness, and decreased joint range of motion [2]. In clinical diagnosis, DOMS symptoms progressively increase after exercise, peak at 2-3 days after exercise, and then dissipate by 5–7 days later [3]. The visual analog scale (VAS) is commonly used to quantify DOMS pain in many studies [4]. The pressure pain threshold (PPT) was demonstrated as reliable for measuring the pain threshold [5] and was also a clinical marker to assess DOMS [6]. Other assessments, such as muscle strength or hematology analysis, were also used to assess the recovery of DOMS [3].

Although the progression of DOMS is not a serious problem, the discomfort can affect exercise participation for athletes. Exercise professionals must take particular care during periods of DOMS, as muscle injuries and functional deficiencies may augment the risk of sports injuries, particularly when returning to sports, or advancing training practices [7]. Thus, strategies to minimize DOMS symptoms and facilitating muscle recovery are of great interest to athletes and athletic trainers, with recovery interventions playing a key role in optimizing sport performance [8]. Many conservative treatments, including massage, cryotherapy, ultrasound, and transcutaneous electrical nerve stimulation, are used to decrease DOMS symptoms, but their effects are inconsistent [9]. If necessary, analgesics are used, but they have short-term effects and there is a risk of abuse [10]. Therefore, nonmedicated, complementary therapies for DOMS-related pain relief have gained popularity in recent times.

Acupuncture is a medical procedure of traditional Chinese medicine and is a common complementary therapy for treating DOMS. A previous systematic review has provided evidence for the effects of acupuncture on musculoskeletal disorders [11]. The therapeutic mechanism of acupuncture has been explained, and it is commonly used for musculoskeletal conditions in clinical practice [12]. The application of needle acupuncture to tender points (i.e., Ah-Shi acupuncture point) or other acupuncture points to reduce DOMS symptoms is feasible [9]. However, the evidence for the effects of acupuncture on DOMS is inconsistent. Therefore, the aim of this study was to conduct a systematic review and a meta-analysis to explore the effects of acupuncture on DOMS.

## 2. Methods

### 2.1. Search Strategy and Data Extraction

We conducted the systematic review and meta-analysis in accordance with standard guideline and Preferred Reporting Items for Systematic Reviews and Meta-Analyses (PRISMA) statement [13, 14]. The searching strategy was based on patient, intervention, comparison, outcome, and study type (PICOs) in Table 1. The keywords “DOMS,” “acupuncture,” “therapy,” “muscle soreness,” and “acupuncture” were used to search in the PubMed, EMBASE, Chinese Electronic Periodical Services, Scopus, Google Scholar, and Cochrane Library electronic databases. The search strategy in PubMed was that #1 (“delayed onset muscle soreness” [All Fields] OR “muscle soreness” [All Fields]); #2 (“acupuncture” [ MeSH Terms] OR “acupuncture therapy” [MeSH Terms]); and #3 (#1 AND #2). The search strategy in EMBASE was that #1 (“delayed onset muscle soreness”/exp OR “muscle soreness”); #2 (“acupuncture” OR “delayed onset muscle soreness”); and #3 (#1 AND #2). Experimental studies related to human subjects and published in journals before March 2020 were included. The inclusion criteria were as follows: control study design articles; the participant has received the procedure for inducing DOMS; intervention was acupuncture applying on acupuncture points after inducing DOMS; and the controls used were sham or no acupuncture, and the outcomes in the follow-up were recorded. The exclusion criteria were as follows: articles of case reports, review studies, and experimental groups received interventions of laser acupuncture, electroacupuncture, or another traditional Chinese medicine intervention.

First, the included abstracts were reviewed and screened by two specialists in sports medicine with more than 5 years of experience. Second, suitable articles were selected, and the full texts were collected and reviewed carefully by the two specialists. The detailed data from each article were independently extracted. The author's name, year of publication, number of participants, intervention, and results were reviewed and collected. The items (i.e., affected muscles, groups, acupuncture points, treatment sessions, assessments, assessment times, and outcomes) in the included articles were collated and analyzed on the basis of the data recorded by the two specialists. Finally, the data from the included articles were analyzed by meta-analysis in accordance with outcome data integrity.

### 2.2. Methodological Quality Assessment

The Jadad scale, which included questionnaire items of randomization, blinding, withdrawals, and dropouts, was used to assess the methodological quality of articles [15]. The scale was scored between 0 and 5, with higher scores indicting higher article quality. For each study, the score was assessed independently with the advice of the two specialists.

### 2.3. Quality Assessment

The Cochrane risk of bias tool was used to assess the bias risk in each included study and judge the methodological quality of the article via seven individual elements. The results of “low bias,” “unclear,” or “high bias” were presented for each element. For each study, the score was assessed independently with the advice of the two specialists.

### 2.4. Statistical Analysis

The outcomes of the included articles were collected and meta-analyzed using MedCalc software (MedCalc, Mariakerke, Belgium). The results of the acupuncture group were compared with those of the sham acupuncture and control groups. The assessments and assessment times were subgrouped to compare the standardized mean differences (SMDs) between the groups. The SMDs and 95% confidence intervals (CIs) were estimated from the means and standard deviations of the results of the included articles. The VAS decrease of DOMS was represented as the improvement of pain. When the acupuncture group was compared with the sham and control groups, a negative SMD favoured the acupuncture group, and the outcomes of the acupuncture group were proved. Heterogeneity was tested by the Q-statistic test. The result was considered a significant heterogeneity for *P* < 0.05 or *I*^2^ > 50%, and a total random-effects model was used to predict the total effects of the acupuncture, sham acupuncture, and control groups. A total fixed-effects model was used when significant homogeneity occurred, and a total random-effects model was used in a significant heterogeneity.

A file drawer analysis was used to assess the publication bias of the included articles. The total effects of various assessments and the effects in various assessment times were used for subgroup analysis to identify the effects of acupuncture on DOMS. Cohen's rule was used to grade the effect size; SMDs of 0.01–0.2, 0.2–0.5, 0.5–0.8, and >0.8 indicated very small, small, moderate, and large effect sizes, respectively [16].

## 3. Results

**Figure 1:**
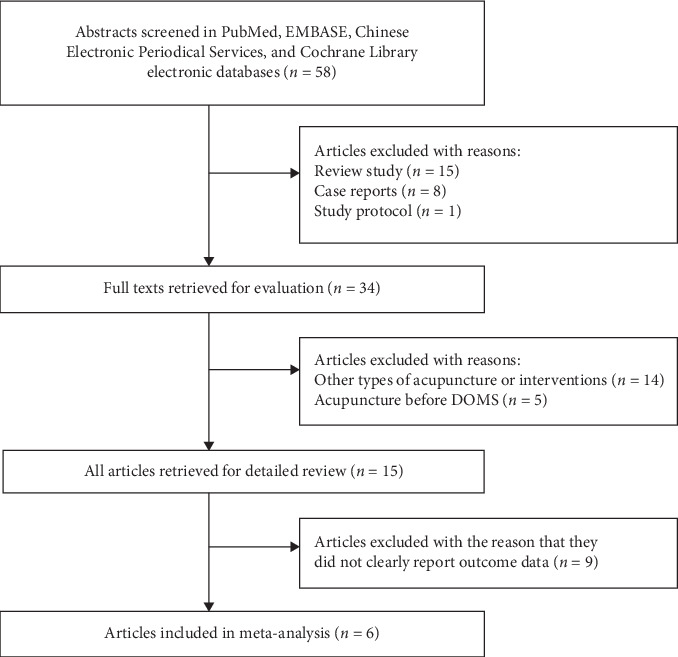
Flow diagram of article selection process.

**Figure 2:**
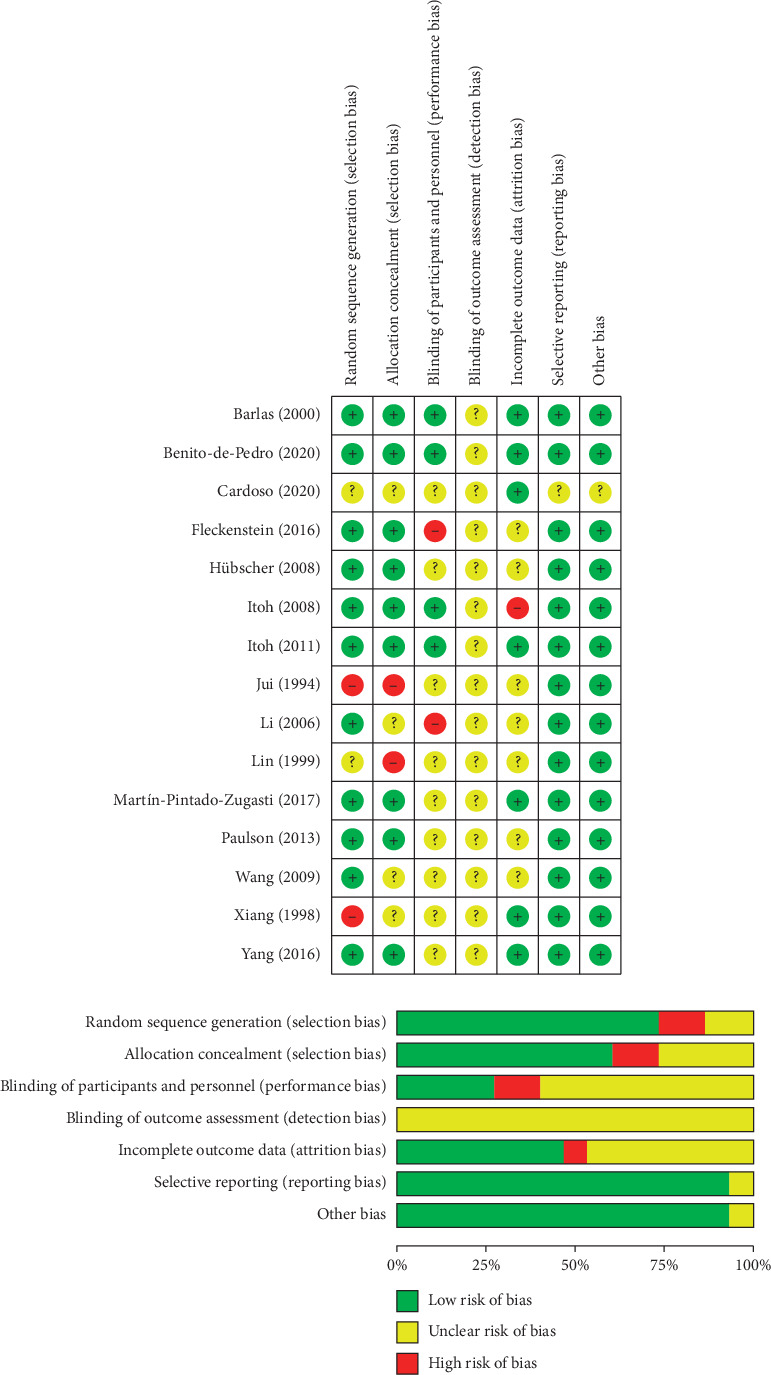
Summary of bias risk.

After searching the electronic databases, 58 abstracts were included. Following discussions with the specialists, 1 study protocol, 8 case reports, and 15 review studies were excluded. Thus, 34 studies of interventions for DOMS in humans remained, and the full texts were reviewed. Among these 34 studies, 14 articles researched other nontraditional types of acupuncture or interventions in traditional Chinese medicine, and five articles used acupuncture before DOMS in an experimental group were excluded. Finally, 15 studies about acupuncture for DOMS were reviewed (Figure 1) [17–31]. The studies included seven English and three non-English articles. The risk of bias for all the 15 articles assessed and the results are summarized in Figure 2. In the included articles, there were 7 two-arm trials, 7 three-arm trials, and 1 four-arm trial. Sixteen acupuncture groups (*n* = 192), 10 sham groups (*n* = 132), and 13 control groups (*n* = 153) were analyzed, as reported in Table 2.

### 3.1. Acupuncture Treatment

In the acupuncture group, the acupuncture points were chosen in accordance with the induced DOMS muscle and tenderness symptoms. However, Barlas et al. [28] applied needles to acupuncture points for one group and to tender points (Ah-Shi acupuncture points) for another group. Benito-de-Pedro et al. and Martín-Pintado-Zugasti et al. used dry needling on tender points via the Ah-Shi acupuncture point procedure for DOMS [18, 20]. Itoh et al. [25] applied needles on tender points via the Ah-Shi acupuncture point procedure, which is based on clinical acupuncture manipulation. Based on the meridian theory of traditional Chinese medicine, needles were inserted into the acupuncture points for 3–30 mins, and the participants felt the “de-qi” sensation. The acupuncture treatment sessions were 1 time per day, or among 1 day to 2 weeks (Table 2). For the sham acupuncture groups in all the reviewed studies, acupuncture was applied to a sham acupuncture point (near the correct acupuncture point). The control groups did not receive any treatments. Both groups were compared with the acupuncture groups to analyze the effects.

### 3.2. Assessment Tools

#### 3.2.1. Pain

The VAS was used to assess the intensity of muscle pain [17, 19–21, 25, 26, 28]. No pain received a score of 0, and maximum pain received a score of 10. The soreness level of DOMS muscle during preforming functional activity was assessed by VAS. The PPT was also measured to assess pain, with a minimum force applied to the affected muscle with DOMS [17–19, 23, 26, 28, 29, 31]. PPT was applied to selected points with a pressure increase rate of 1 kg/cm^2^/s until participants felt tenderness.

#### 3.2.2. Muscle Strength

Maximum isometric voluntary force was assessed by the strain-gauge force transducer (ASYS Sporeg, Offenbach, Germany) and was defined as isometric muscle strength [19, 26]. Isokinetic muscle strength, including eccentric and concentric muscle contractions, was evaluated using a Kin-Com dynamometer (Chattecx Corporation, Chattanooga, TN) [31].

#### 3.2.3. Biochemical Analysis

Blood samples of the participants were taken, and their serums were separated. The levels of luteinizing hormone, testosterone, cortisol, and serum creatine kinase were analyzed [24, 27, 29–31].

#### 3.2.4. Joint Range of Motion

Joint range of motion was measured using a goniometer via passive joint motion, which was limited by antagonist muscle of DOMS [28].

#### 3.2.5. Psychophysiological Response

Thermographic measurement was performed with a thermographic camera to assess skin temperature and blood circulation, originated by autonomic nervous system stimulation [18]. Psychological factors, such fear and anxiety for pain or kinesiophobia, on the outcomes of acupuncture for DOMS were also measured by psychological questionnaires [20]. In the study by Paulson et al., skin conductance and skin temperature were used as indicators of sympathetic nervous system activation. They were assessed using FlexComp Infiniti SC-Flex/Pro SC sensors (Thought Technology, New York, USA) [22].

### 3.3. Study Outcomes

In Table 3, analyses of all the included articles revealed that the decrease of VAS appeared after acupuncture and then gradually declined during the treatment sessions. In particular, pain was significantly decreased on day 3 (*P* < 0.05) [17, 20, 21, 25, 26, 29]. Joint motion and muscle strength were also improved during acupuncture treatment sessions, but there were no significant differences [19, 26, 28, 31]. Four of the included articles had found that PPT in the acupuncture group was significantly improved compared to sham or control groups (*P* < 0.05) [18, 23, 29, 31]. But, in the four articles, the outcomes on PPT did improve significantly (*P* > 0.05) [17, 19, 26, 28]. Paulson et al. [22] observed increased skin conductance and decreased skin temperature after acupuncture, and the differences were significant (*P* < 0.05). However, biochemical analysis of blood (i.e., luteinizing hormone, testosterone, cortisol, and serum creatine kinase) revealed no significant differences between acupuncture and control groups [24, 27, 29–31]. Barlas et al. [28] noted no increase in joint motion in the acupuncture group after treatment. The changes on psychological measure and skin temperature were also noted at postacupuncture for DOMS [18, 20, 22].

### 3.4. Results of Meta-Analysis

When inducing DOMS, the VAS data from the articles were integrated after acupuncture. In the follow-up, VAS was collected to perform subgroup analysis. Nine articles were excluded with the reason that they did not clearly report outcome data, i.e., unavailable data [18, 22–24, 27, 29, 30], and missing data [26, 31]. Therefore, the data from the six articles were included for meta-analysis. The Jadad scales of the studies were in the range of 2–5, and they had moderate methodological quality (average score = 3.13 ± 1.18, Table 2).

**Figure 3:**
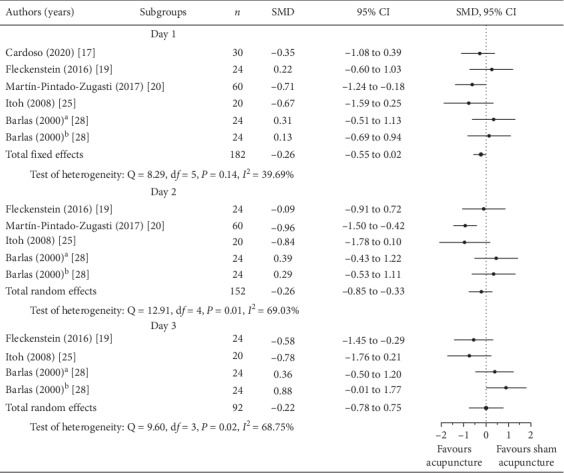
Forest plots; subgroup analysis of pain at days 1, 2, and 3 (acupuncture vs. sham acupuncture groups; ^a^acupuncture on P2, CO11, LU5, and CO4 acupuncture points; ^b^acupuncture on Ah-Shi acupuncture point).

Rosenthal's fail-safe number was used to analyze the publication bias in meta-analysis [32]. Based on the outcomes of the included articles, the tolerance level of 85 was lower than the fail-safe number of 225. Therefore, the publication bias could not affect the results of meta-analysis. In three articles, the VAS was reported on days 1–3 after inducing DOMS. The effects of acupuncture on DOMS could be inferred by comparing the SMDs of the acupuncture groups with those of the sham acupuncture or control groups. The VASs on days 1–3 were compared with acupuncture and sham acupuncture groups in Figure 3. The results of meta-analyses revealed that there were homogeneity in VAS on day 1 (95% CI for *I*^2^ = 0.01∼76.07, *P*_heterogeneity_ = 0.14) and heterogeneity on days 2 and 3 between the groups (95% CI for *I*^2^ = 20.48∼87.94, *P*_heterogeneity_ = 0.01; 95% CI for *I*^2^ = 18.10∼89.92, *P*_heterogeneity_ = 0.01, respectively). The result of meta-analysis for decrease of VAS on days 1–3 was in favour of acupuncture, and the total effects demonstrated very-small-to-small effect sizes on day 1 (total SMD = −0.26, 95% CI = −0.55∼0.02, *P*=0.07), day 2 (total SMD = −0.26, 95% CI = −0.85∼0.33, *P*=0.38), and day 3 (total SMD = −0.02; 95% CI = −0.78∼0.75, *P*=0.95). Compared with sham acupuncture, the effects of pain relief from acupuncture on DOMS were very small to small, with nonsignificant differences between the two groups (*P* > 0.05).

**Figure 4:**
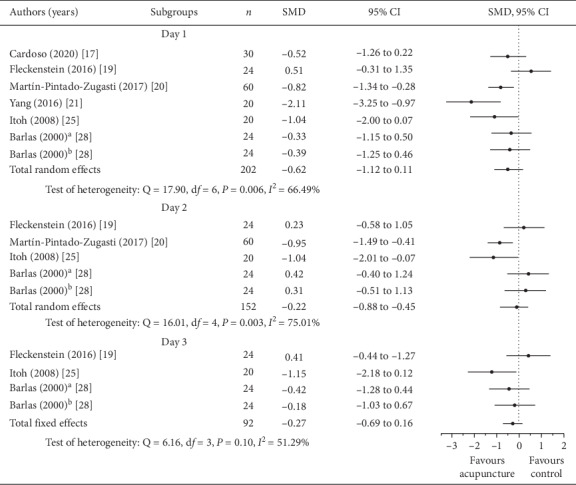
Forest plots; subgroup analysis of pain at days 1, 2, and 3 (acupuncture vs. control group; ^a^acupuncture on P2, CO11, LU5, and CO4 acupuncture points; ^b^acupuncture on Ah-Shi acupuncture point).

The effects of VAS decrease were observed by comparing the acupuncture and control groups in Figure 4. Decrease of VAS on days 1 and 2 (95% CI for *I*^2^ = 25.17∼84.99, *P*_heterogeneity_ = 0.006; 95% CI for *I*^2^ = 38.40∼89.86, *P*_heterogeneity_ = 0.003, respectively) had significant heterogeneity in both groups, but those on day 3 did not have heterogeneity (*P*_heterogeneity_ = 0.08). Comparing both groups, the SMD on day 1 was significant for VAS decrease in favour of acupuncture (total SMD = −0.62; 95% CI = −1.12∼−0.11, *P*=0.01), but there were no significant differences on day 2 and 3 (total SMD = −0,22; 95% CI = −0.88∼0.45, *P*=0.51; total SMD = −0.27; 95% CI = −0.69∼0.16, *P*=0.18, respectively). The effect on pain relief of DOMS was in favour of acupuncture, and the meta-analysis on day 1 revealed that the acupuncture group had a moderate effect when compared to the control group (*P* < 0.05). The acupuncture group also had small effect for DOMS pain relief on days 2 and 3, but there were no significant differences (*P* > 0.05).

## 4. Discussion

The onset of DOMS occurred between 2 and 3 days after the exercise and decreased progressively by 5–7 days, indicating the utility of DOMS as a method of determining the effects of acupuncture on muscle pain. Athletes incorporate muscle strength to optimise sport performance and gain a competitive edge [33]. However, intensified training may also cause DOMS that can last for several days after exercise, which may affect neuromuscular control, leading to a decreased explosive muscular force and resulting in an increased risk of sports injuries [34]. Garlanger et al. [35] found that athletes with DOMS often recommended acupuncture to other athletes after receiving it. The acupuncture in traditional Chinese medicine could be a complementary therapy, and the acupuncture experience was accepted in athletes. However, the effects of acupuncture on pain relief for DOMS require further evidence. Through our systematic review, we found that acupuncture reduced the level of DOMS in twelve studies [17, 18, 20–23, 25, 26, 28–31], but three studies did not report any benefits of acupuncture on DOMS [19, 24, 27]. Although the improvement of PPT and muscle strength on DOMS after receiving acupuncture was found, a reason for insufficient data leads to the difficultly conducting meta-analysis. So, only the effect sizes of VAS were analyzed at days 1–3 between acupuncture and sham acupuncture or control. The meta-analysis revealed no evidence that acupuncture decreased pain more effectively than sham acupuncture. When comparing DOMS measures between acupuncture and control, the effect size on pain relief was small to moderate at days 1–3. It implies that acupuncture for DOMS seems to be used in pain relief at post-DOMS compared to nonuse of acupuncture.

Three of the included articles supported the analgesic effect of acupuncture on DOMS, suggesting that acupuncture is effective in managing pain in DOMS [25, 26, 29]. Although the mechanism of acupuncture on DOMS remains unknown, the neurostimulation and Chinese meridian theory were used to explain the analgesic effect. Okada and Kawakita [36] argued that the pain-relieving mechanism of acupuncture is a diffuse noxious inhibitory control phenomenon, which is why acupuncture can decrease the muscle pain associated with DOMS. Fleckenstein [37] indicated that acupuncture on skin surface caused physical stimulation (i.e., the “de-qi” sensation). This neural stimulation could activate the spino-bulbo-spinal circuit and inhibit wide-dynamic-range neurons, resulting in a short-term analgesic effect [38]. Barlas et al. [28] thought that the manual twisting of needles at approximately 1 Hz is used to obtain a “de-qi” sensation. The physical stimulation with low frequencies could stimulate active endogenous opioid systems and reduce the pain sensation after acupuncture [39]. Wang [24] supported that acupuncture for DOMS could relax muscles and have an analgesic effect. It also affects the activation of anti-free radicals and improves hypothalamic-pituitary-gonadal disorders [40]. It is also possible that the desensitisation of the nociceptive afferent from muscular inflammation of DOMS could lead to relieving muscle guarding, which may ameliorate the limitation of joint range of motion caused by DOMS. However, the evidence from a previous study reported no differences between acupuncture and sham on joint range of motion [24]. Thus, if muscle guarding was not relieved from acupuncture, it is impossible to relax the muscles, which implies little effect of analgesia. Whilst the included studies demonstrated that the analgesic effect occurred during acupuncture treatment sessions after inducing DOMS [20, 25, 26, 29], the very-small-small effect sizes found in the meta-analysis for pain relief limited our confidence on the benefits of acupuncture for DOMS.

Our meta-analysis revealed small-to-moderate analgesic effects of acupuncture on DOMS, especially on day 1 (total SMD = −0.62; 95% CI = −1.12∼0.11, *P* < 0.05) by comparing the acupuncture and control groups. However, the evidence on effects of pain relief for acupuncture was still weak in the current study because follow-up data of DOMS recovery were insufficient to perform a meta-analysis. In particular, the meta-analysis revealed very-small-to-small effects of acupuncture on DOMS by comparing the acupuncture and sham acupuncture groups, with no significant difference (*P* > 0.05). Therefore, acupuncture and sham acupuncture had similar outcomes for DOMS. The treatment-related nonspecific effect, which is a psychological effect, occurred because a patient was touched or received acupuncture on their skin [41, 42]. The wide variety of factors involved in acupuncture treatment sessions, including acupuncture points, treatment times, frequency, and duration, were applied to DOMS in the included articles. Barlas et al. [28] thought that a number of acupuncture treatment sessions were required to achieve analgesic effects, and suitable treatment parameters for DOMS were needed to warrant more investigations.

In previous studies, tender points (i.e., the Ah-Shi acupuncture points) were chosen for treating DOMS with acupuncture [20, 23, 25, 28]. DOMS causes muscle soreness and pain, and acupuncture on tender points can address the muscular condition. Acupuncture stimulation of tender points could activate the sensitized polymodal-type receptors of the muscle to reduce pain [43]. Acupuncture on manifold acupuncture points, which combine meridian acupuncture points and Ah-Shi points, were considered for the treatment of DOMS [19, 26, 29]. Combining the acupuncture points for DOMS could be another choice for physicians. In our review, we found another treatment, i.e., “dry needling,” that is similar to acupuncture, and it is gaining attention for the treatment myofascial trigger points [44, 45]. However, “dry needling” is a different proper noun and clinical procedure, so we excluded these articles from our review.

Fleckenstein et al. [19] indicated that DOMS is a self-limiting motion process that is strongly influenced by local muscle nociceptors. The neural mechanisms of DOMS and chronic pain, which may involve the neuroplasticity, are different. Moreover, the physiological mechanisms that cause the positive effects of acupuncture on muscle pain in DOMS require clarification [46, 47]. In addition to a subjective VAS, the assessments of PPT, muscle strength, and joint motion provided further evidence for objective evaluations. Paulson and Shay [22] attempted to demonstrate that improved sympathetic nervous system activity can explain the effects of acupuncture on DOMS. Therefore, multifaceted and high-quality studies should focus on the benefits of acupuncture; thus, the beneficial effects of acupuncture on DOMS may be proved in the future.

As with all systematic reviews, several limitations should be identified. First, a lack of the same specific assessments for DOMS could be classified in the subgroup analysis. Second, the outcomes of some included studies were presented as figures, showing the changes in assessments in the treatment session. Therefore, it is difficult to extract data, resulting in insufficient data form the assessments, such as muscle strength or PPT. Hence, the meta-analysis could only be performed using a limited number of studies.

## 5. Conclusion

Our systematic review and meta-analysis revealed that acupuncture had very-small-to-small and small-to-moderate effects on pain relief for DOMS when compared with sham and no acupuncture, respectively. The evidence for the benefit of acupuncture to relieve the symptoms of DOMS was still weak. Based on our review, limited findings of acupuncture for DOMS were concluded through insufficient data due to a lack of high-quality and well-reported articles.

## Figures and Tables

**Table 1:** Items of PICOs in our study.

Items	Description
Patient or problem	Human subjects with DOMS
Intervention	Acupuncture
Comparison	Sham or no acupuncture
Outcome	Pain or physiological recovery
Study type	Control study design

PICOs: patient, intervention, comparison, outcome, and study type.

**Table 2:** Summary of affected muscles, treatment programs, and study quality in the included articles.

Author (years)	Affected muscles	Groups (*n*)	Acupuncture points	Treatment session	Quality
Cardoso et al. (2020) [17]	Quadriceps	Acupuncture (*n* = 15)	LR3, ST34, ST36	5 insertions; 1 time	4
		Sham (*n* = 15)	Sham acupuncture points	5 insertions; 1 time	
		Control (*n* = 15)	No acupuncture	No treatment	
Benito-de-Pedro et al. (2020) [18]	Triceps surae	Acupuncture (*n* = 17)	Ah-Shi	8–10 insertions; 1 time	5
		Sham (*n* = 17)	Sham acupuncture points	90 sec	
Fleckenstein et al. (2016) [19]	Biceps brachii	Acupuncture (*n* = 12)	LI4, LI11, LU3, LU5, GB34, SP10, Ah-Shi	De-qi; 15 mins; 1time/day; 3 days	5
		Sham (*n* = 12)	Sham acupuncture points	15 mins; 1time/day; 3 days	
		Control (*n* = 12)	No acupuncture	No treatment	
Martín-Pintado-Zugasti et al. (2017) [20]	Upper trapezius	Acupuncture (*n* = 30)	Ah-Shi	4 mins, 1 time	2
		Sham (*n* = 30)	Sham acupuncture points	4 mins, 1 time	
		Control (*n* = 30)	No acupuncture	No treatment	
Yang (2016) [21]	Quadriceps	Acupuncture (*n* = 10)	Ah-Shi, SP10, SP6, GB34, GB30, BL23	25 mins; 1 time	3
		Control (*n* = 10)	No acupuncture	No treatment	
Paulson and Shay (2013) [22]	Biceps brachii	Acupuncture (*n* = 12)	LI4, LI10, LI11, TE5	De-qi; 15 mins; 1 time	3
		Sham (*n* = 12)	Sham acupuncture points	15 mins; 1 time	
		Control (*n* = 12)	No acupuncture	No treatment	
Itoh et al. (2011) [23]	Extensor digital	Acupuncture (*n* = 6)	Ah-Shi	5 mins; 3 times	3
		Sham (*n* = 6)	Sham acupuncture points	5 mins; 3 times	
		Control (*n* = 6)	No acupuncture	No treatment	
Wang (2009) [24]	Quadriceps	Acupuncture (*n* = 5)	ST36, RN8	20 mins, 1 time/day, 2 weeks	2
		Control (*n* = 5)	No acupuncture	No treatment	
Itoh et al. (2008) [25]	Biceps brachii	Acupuncture (*n* = 10)	Ah-Shi	De-qi; 10 mins; 1 time	5
		Sham (*n* = 10)	Sham acupuncture points	10 mins; 1 time	
		Control (*n* = 10)	No acupuncture	No treatment	
		Sham (*n* = 10)	Sham acupuncture points	3 mins, 1 time/day; 4 days	
Hübscher et al. (2008)[26]	Biceps brachii	Acupuncture (*n* = 7)	GB34, LU3, LU5, LI11, SP10, Ah-Shi	De-qi; 15 mins; 1 time/day; 3 days	4
		Sham (*n* = 8)	Sham acupuncture points	15 mins; 1 time/day; 3 days	
		Control (*n* = 7)	No acupuncture	No treatment	
Li and Zhai (2006) [27]	Quadriceps, gastrocnemius, solus	Acupuncture (*n* = 14)	ST36, GB34, BL20, BL23, BL11, BL12, BL25, BL57, Ah-Shi	De-qi; 30 mins, 1 time/day, 1 week	2
		Control (*n* = 14)	No acupuncture	Non-treatment	
Barlas et al. (2000) [28]	Biceps brachii	Acupuncture (*n* = 12)	P2, CO11, LU5,CO4	De-qi; 5 mins; 1 time/day; 5 days	3
		Acupuncture (*n* = 12)	Ah-Shi	De-qi; 5 mins; 1 time/day; 5 days	
		Sham (*n* = 12)	Sham acupuncture points	5 mins; 1 time/day; 5 days	
		Control (*n* = 12)	No acupuncture	No treatment	
Lin and Yang (1999) [29]	Biceps brachii	Acupuncture (*n* = 10)	GB34, LU3, LU5, LI11, Ah-Shi	De-qi; 20 mins; 1 time/day; 5 days	2
		Control (*n* = 10)	No acupuncture	No treatment	
Xiang et al. (1998) [30]	Quadriceps	Acupuncture (*n* = 10)	PC6, GB30, ST36, GB34, BL57	30 mins; 1 time	2
		Control (*n* = 10)	No acupuncture	No treatment	
Jui (1994) [31]	Quadriceps	Acupuncture (*n* = 10)	SP10, GB31, ST36	3 mins, 1 time/day; 4 days	2
		Sham (*n* = 10)	Sham acupuncture points	3 mins, 1 time/day; 4 days	

BL11, Dazhu; BL12, Fengmen; BL20, Pishu; BL23, Shenshu; BL25, Dachangshu; BL56, Chengjin; BL57, Chengshan; CO4, Wei; CO11, Yidan; GB30, Huantiao; GB31, Fengshi; GB34, Yanglingquan; LI4, Hegu; LI10, Shousanli; LI11, Quchi; LR3, Taichong; LU3, Tianfu; LU5, Chize; P2, Erbeifei; PC6, Neiguan; RN8, Shengue; SP6, Sanyinjiao; SP9, Yinlingquan; SP10, Xuehai; ST34, Liangqiu; ST36, Zusanli; and TE5, Waiguan.

**Table 3:** Summary of assessments, assessment times, and outcomes in the included articles.

Author (years)	Assessments	Assessment times	Outcomes
Cardoso et al. (2020) [17]	VAS, PPT	Pre-post acupuncture, day 1	Significant decrease in VAS at postacupuncture among the groups^*∗*^
Benito-de-Pedro et al. (2020) [18]	PPT, thermographic measurement	Pre-post acupuncture	Improved PPT at postacupuncture^*∗*^ A significant difference in PPT between the groups
Fleckenstein et al. (2016) [19]	VAS, PPT, muscle strength	Preacupuncture, days 1, 2, and 3	No significant differences in all assessments at days 1–3 among the groups
Martín-Pintado-Zugasti et al. (2017) [20]	VAS, psychological measure	Postacupuncture, days 0.5, 1, and 2	Significant decrease in VAS over time among the groups^*∗*^
Yang (2016) [21]	VAS	Postacupuncture, day 1	Significant differences in VAS between the groups^*∗*^
Paulson and Shay (2013) [22]	Sympathetic nervous system responses	Pre-post acupuncture	Increased skin conductance and decreased skin temperature at postacupuncture^*∗*^
Itoh et al. (2011) [23]	PPT	Pre-post acupuncture, day 2	Increased PPT at postacupuncture and day 2^*∗*^
Wang (2009) [24]	Serum creatine kinase, testosterone	Pre-post treatment session	Increased level of serum creatine kinase at posttreatment session No significant differences in testosterone level between the groups
Itoh et al. (2008) [25]	VAS	Pre-post acupuncture, days 1, 2, 3, and 7	Decreased VAS at postacupuncture^*∗*^ A significant decrease in VAS at day 3 among the groups^*∗*^
Hübscher et al. (2008) [26]	VAS, PPT, muscle strength	Pre-post acupuncture, days 1, 2, and 3	Decreased VAS and increased PPT and muscle strength at days 2-3 A significant decrease in VAS at day 3 among the groups^*∗*^
Li and Zhai (2006) [27]	Luteinizing hormone, testosterone, cortisol	Pre-post treatment session	No significant differences in levels of luteinizing hormone, testosterone, and cortisol between the groups
Barlas et al. (2000) [28]	VAS, PPT, joint range of motion	Pre-post acupuncture, days 1, 2, 3, 4, and 5	A significant decrease in VAS at days 1–5 among the groups^*∗*^
Lin and Yang (1999) [29]	PPT, serum creatine kinase	Preacupuncture, days 1, 2, and 3	A significant decrease in VAS at day 3 among the groups^*∗*^
Xiang et al. (1998) [30]	Serum creatine kinase	Pre-post treatment session	Decreased levels of serum creatine kinase at postacupuncture^*∗*^
Jui (1994) [31]	PPT, muscle strength, serum creatine kinase	Preacupuncture, days 1, 2, and 3	A significant improvement in muscle strength at day 3 and in PPT at day 2 between the groups^*∗*^ No significant differences in the levels of serum creatine kinase between the groups

^*∗*^ *P* < 0.05, comparison between group differences.

## Data Availability

The data used to support the findings of this study are included within the article.
